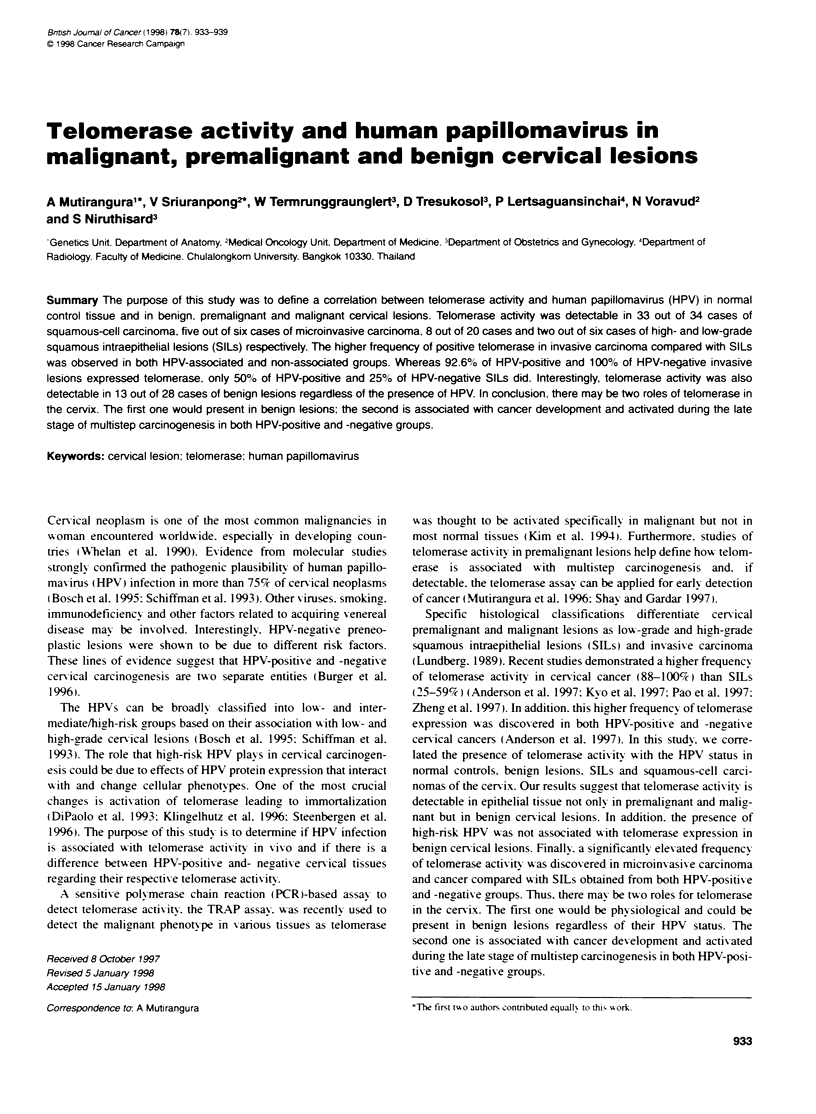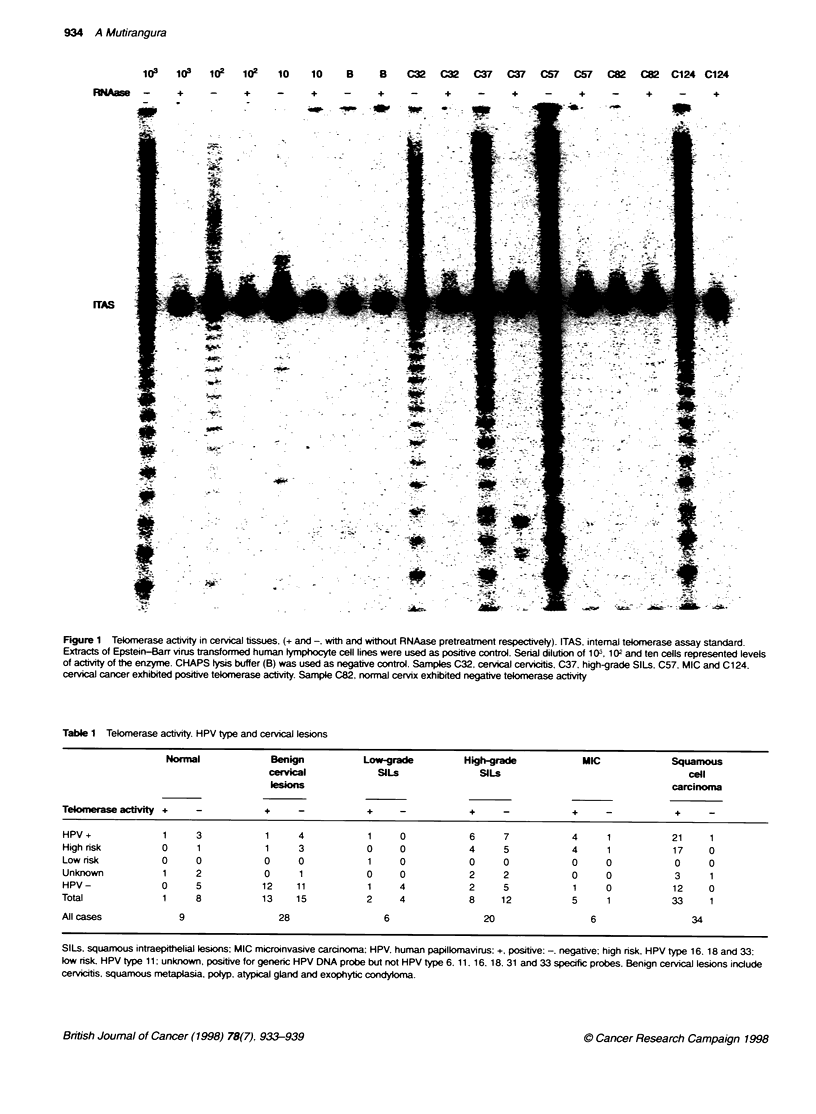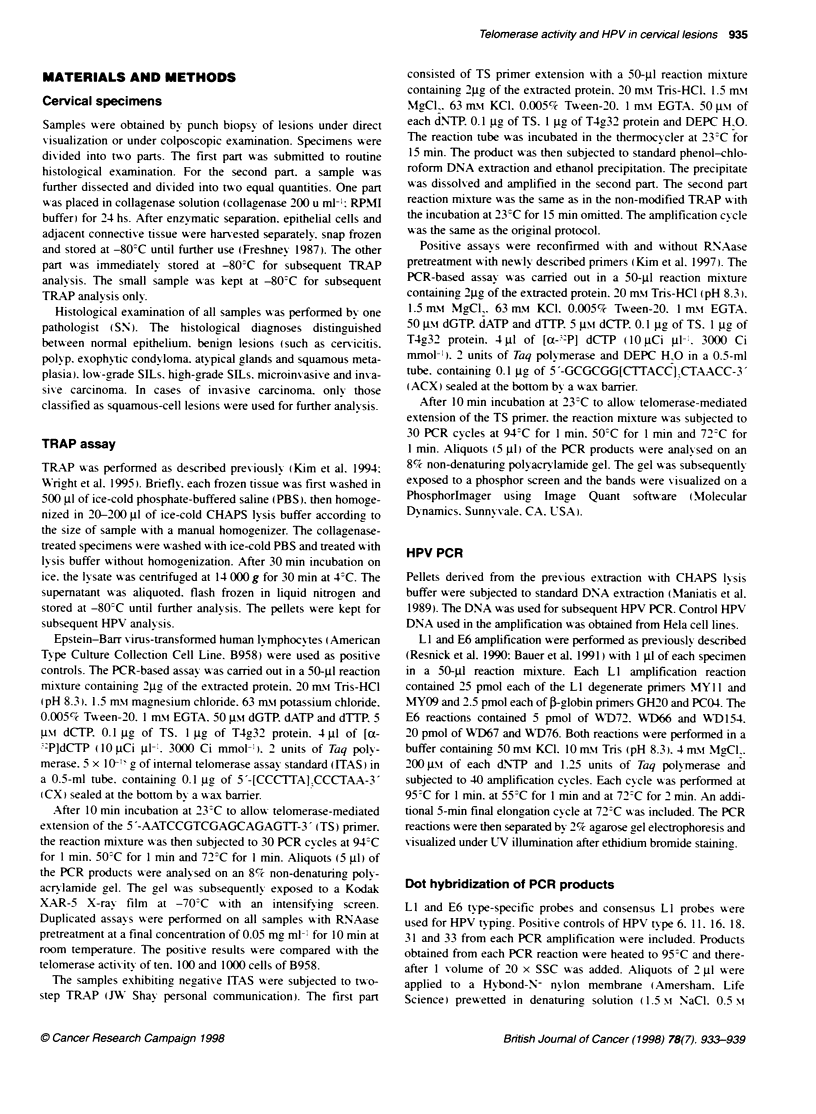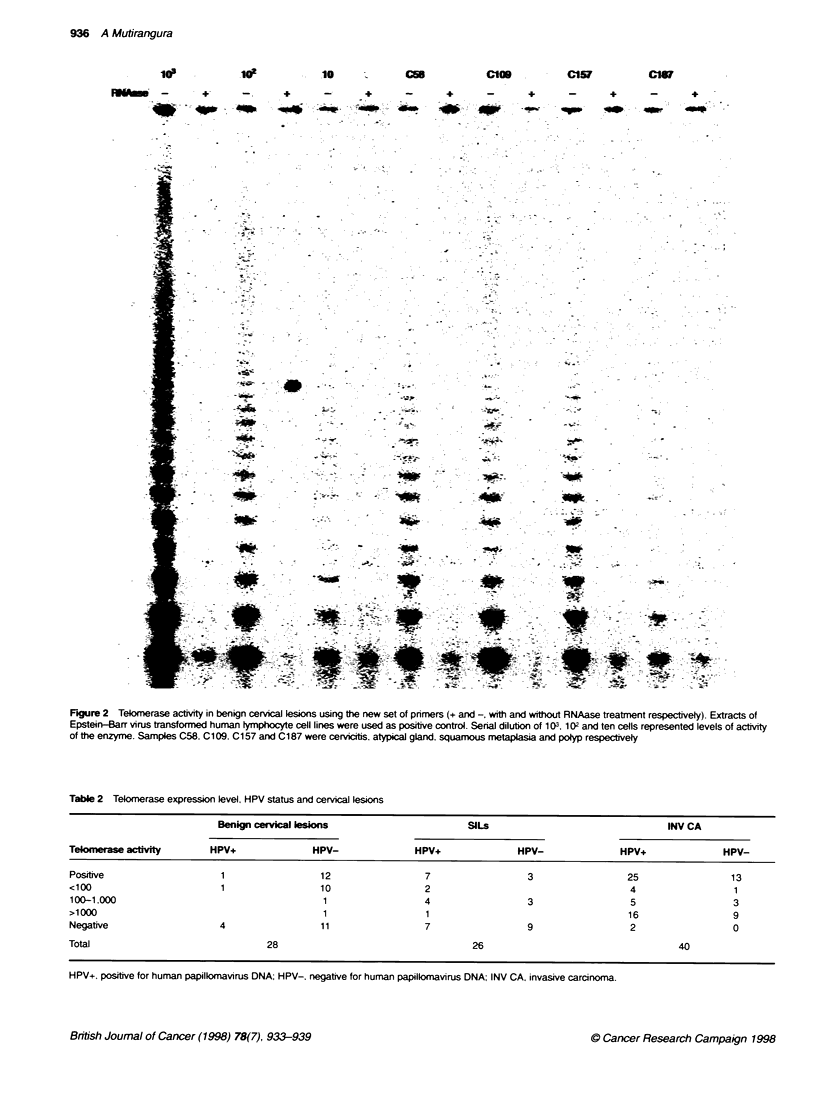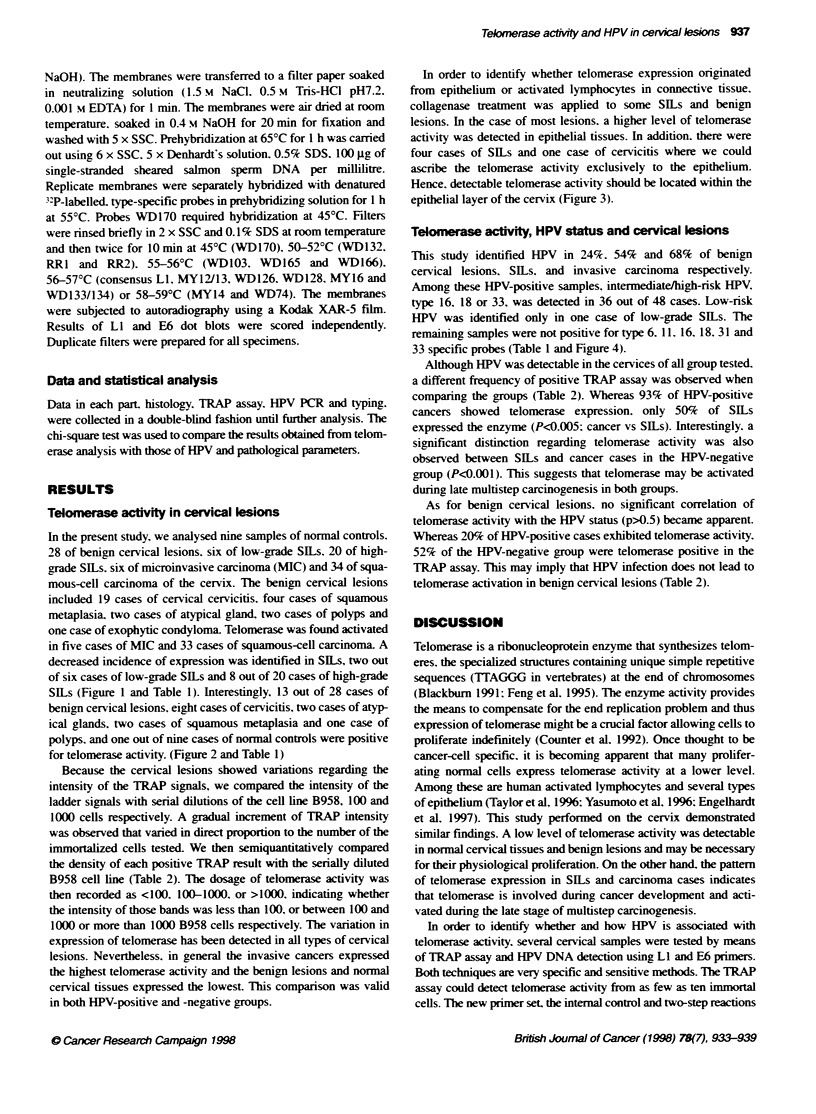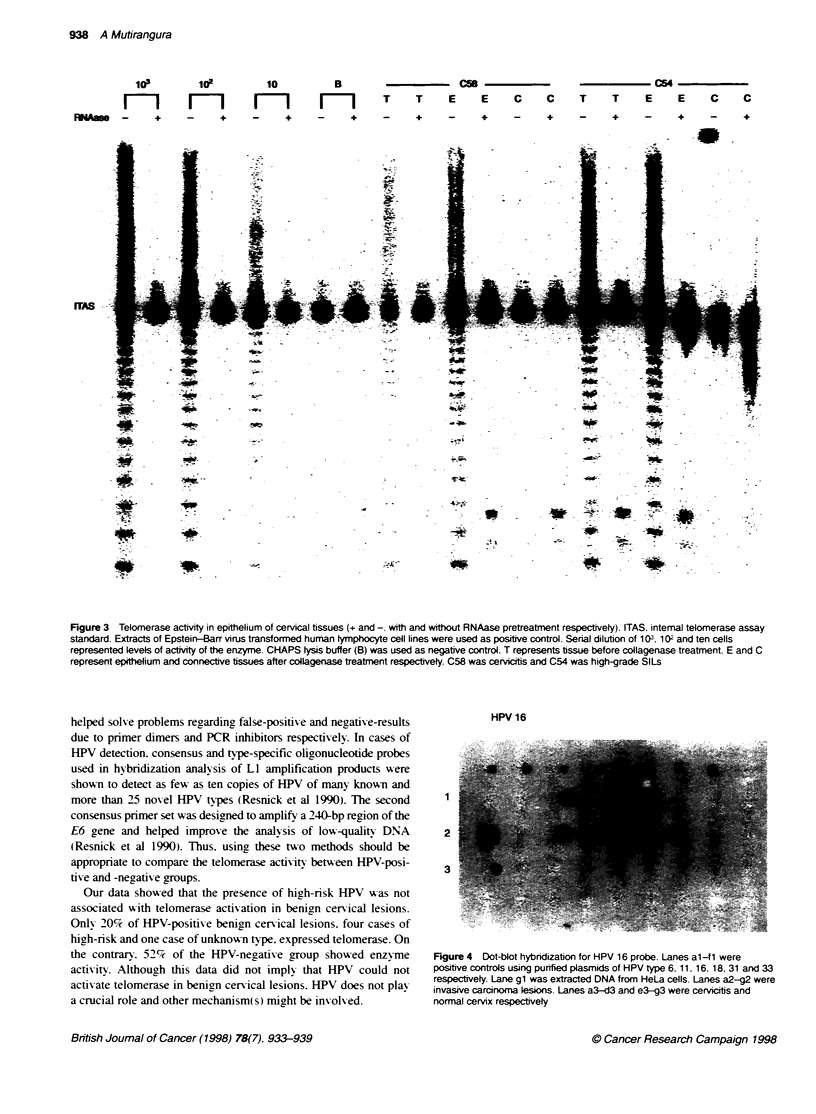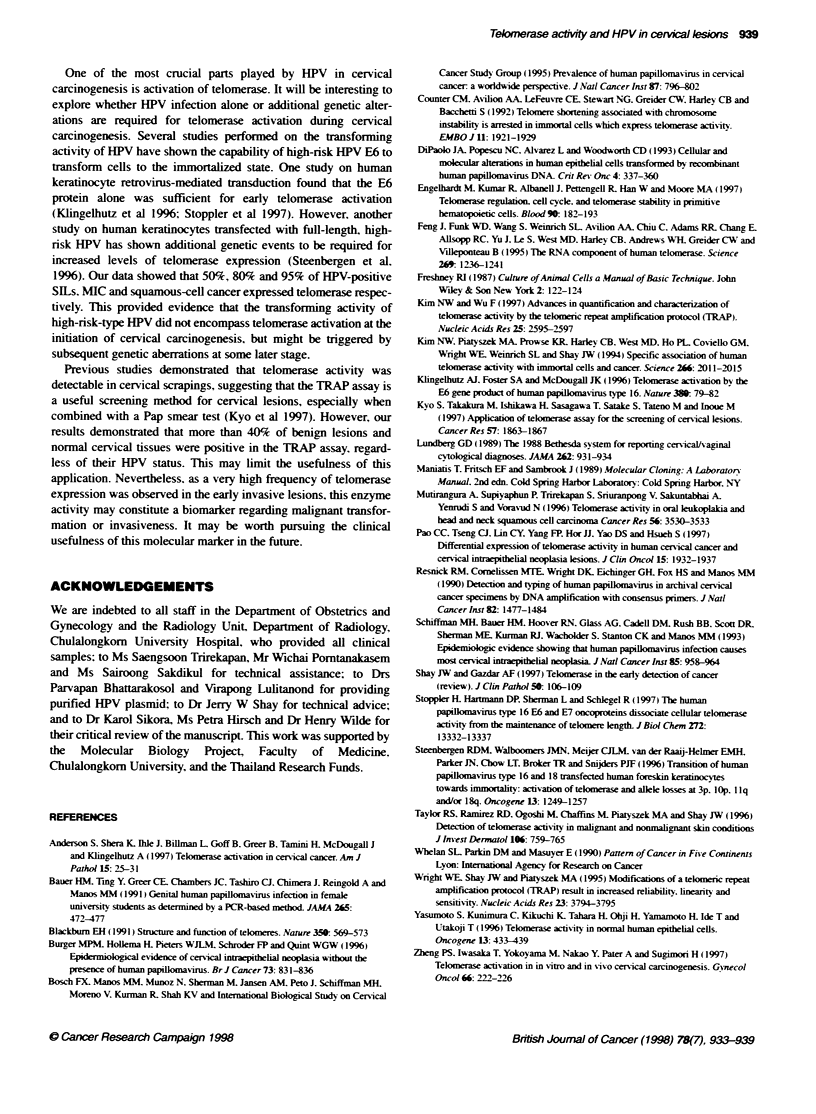# Telomerase activity and human papillomavirus in malignant, premalignant and benign cervical lesions.

**DOI:** 10.1038/bjc.1998.604

**Published:** 1998-10

**Authors:** A. Mutirangura, V. Sriuranpong, W. Termrunggraunglert, D. Tresukosol, P. Lertsaguansinchai, N. Voravud, S. Niruthisard

**Affiliations:** Department of Anatomy, Faculty of Medicine, Chulalongkorn University, Bangkok, Thailand.

## Abstract

**Images:**


British Journal of Cancer (1998) 78(7), 933–939
© 1998 Cancer Research Campaign

### Telomerase activity and human papillomavirus in malignant, premalignant and benign cervical lesions

**A Mutirangura[1*], V Sriuranpong[2*], W Termrunggraunglert[3], D Tresukosol[3], P Lertsaguansinchai[4], N Voravud[2] and S Niruthisard[3]**

[1]Genetics Unit, Department of Anatomy, [2]Medical Oncology Unit, Department of Medicine, [3]Department of Obstetrics and Gynecology, [4]Department of Radiology, Faculty of Medicine, Chulalongkorn University, Bangkok 10330, Thailand

**Summary** The purpose of this study was to define a correlation between telomerase activity and human papillomavirus (HPV) in normal control tissue and in benign, premalignant and malignant cervical lesions. Telomerase activity was detectable in 33 out of 34 cases of squamous-cell carcinoma, five out of six cases of microinvasive carcinoma, 8 out of 20 cases and two out of six cases of high- and low-grade squamous intraepithelial lesions (SILs) respectively. The higher frequency of positive telomerase in invasive carcinoma compared with SILs was observed in both HPV-associated and non-associated groups. Whereas 92.6% of HPV-positive and 100% of HPV-negative invasive lesions expressed telomerase, only 50% of HPV-positive and 25% of HPV-negative SILs did. Interestingly, telomerase activity was also detectable in 13 out of 28 cases of benign lesions regardless of the presence of HPV. In conclusion, there may be two roles of telomerase in the cervix. The first one would present in benign lesions; the second is associated with cancer development and activated during the late stage of multistep carcinogenesis in both HPV-positive and -negative groups.

**Keywords:** cervical lesion; telomerase; human papillomavirus

Cervical neoplasm is one of the most common malignancies in woman encountered worldwide, especially in developing countries (Whelan et al, 1990). Evidence from molecular studies strongly confirmed the pathogenic plausibility of human papillomavirus (HPV) infection in more than 75% of cervical neoplasms (Bosch et al, 1995; Schiffman et al, 1993). Other viruses, smoking, immunodeficiency and other factors related to acquiring venereal disease may be involved. Interestingly, HPV-negative preneoplastic lesions were shown to be due to different risk factors. These lines of evidence suggest that HPV-positive and -negative cervical carcinogenesis are two separate entities (Burger et al, 1996).

The HPVs can be broadly classified into low- and intermediate/high-risk groups based on their association with low- and high-grade cervical lesions (Bosch et al, 1995; Schiffman et al, 1993). The role that high-risk HPV plays in cervical carcinogenesis could be due to effects of HPV protein expression that interact with and change cellular phenotypes. One of the most crucial changes is activation of telomerase leading to immortalization (DiPaolo et al, 1993; Klingelhutz et al, 1996; Steenbergen et al, 1996). The purpose of this study is to determine if HPV infection is associated with telomerase activity in vivo and if there is a difference between HPV-positive and- negative cervical tissues regarding their respective telomerase activity.

A sensitive polymerase chain reaction (PCR)-based assay to detect telomerase activity, the TRAP assay, was recently used to detect the malignant phenotype in various tissues as telomerase was thought to be activated specifically in malignant but not in most normal tissues (Kim et al, 1994). Furthermore, studies of telomerase activity in premalignant lesions help define how telomerase is associated with multistep carcinogenesis and, if detectable, the telomerase assay can be applied for early detection of cancer (Mutirangura et al, 1996; Shay and Gardar 1997).

Specific histological classifications differentiate cervical premalignant and malignant lesions as low-grade and high-grade squamous intraepithelial lesions (SILs) and invasive carcinoma (Lundberg, 1989). Recent studies demonstrated a higher frequency of telomerase activity in cervical cancer (88–100%) than SILs (25–59%) (Anderson et al, 1997; Kyo et al, 1997; Pao et al, 1997; Zheng et al, 1997). In addition, this higher frequency of telomerase expression was discovered in both HPV-positive and -negative cervical cancers (Anderson et al, 1997). In this study, we correlated the presence of telomerase activity with the HPV status in normal controls, benign lesions, SILs and squamous-cell carcinomas of the cervix. Our results suggest that telomerase activity is detectable in epithelial tissue not only in premalignant and malignant but in benign cervical lesions. In addition, the presence of high-risk HPV was not associated with telomerase expression in benign cervical lesions. Finally, a significantly elevated frequency of telomerase activity was discovered in microinvasive carcinoma and cancer compared with SILs obtained from both HPV-positive and -negative groups. Thus, there may be two roles for telomerase in the cervix. The first one would be physiological and could be present in benign lesions regardless of their HPV status. The second one is associated with cancer development and activated during the late stage of multistep carcinogenesis in both HPV-positive and -negative groups.

*Received 8 October 1997*
*Revised 5 January 1998*
*Accepted 15 January 1998*

*Correspondence to:* A Mutirangura

*The first two authors contributed equally to this work.

**Figure 1** Telomerase activity in cervical tissues, (+ and −, with and without RNAase pretreatment respectively). ITAS, internal telomerase assay standard. Extracts of Epstein–Barr virus transformed human lymphocyte cell lines were used as positive control. Serial dilution of $10^3$, $10^2$ and ten cells represented levels of activity of the enzyme. CHAPS lysis buffer (B) was used as negative control. Samples C32, cervical cervicitis, C37, high-grade SILs, C57, MIC and C124, cervical cancer exhibited positive telomerase activity. Sample C82, normal cervix exhibited negative telomerase activity

**Table 1** Telomerase activity, HPV type and cervical lesions

| | Normal | | Benign cervical lesions | | Low-grade SILs | | High-grade SILs | | MIC | | Squamous cell carcinoma | |
|---|---|---|---|---|---|---|---|---|---|---|---|---|
| **Telomerase activity** | + | − | + | − | + | − | + | − | + | − | + | − |
| HPV + | 1 | 3 | 1 | 4 | 1 | 0 | 6 | 7 | 4 | 1 | 21 | 1 |
| High risk | 0 | 1 | 1 | 3 | 0 | 0 | 4 | 5 | 4 | 1 | 17 | 0 |
| Low risk | 0 | 0 | 0 | 0 | 1 | 0 | 0 | 0 | 0 | 0 | 0 | 0 |
| Unknown | 1 | 2 | 0 | 1 | 0 | 0 | 2 | 2 | 0 | 0 | 3 | 1 |
| HPV − | 0 | 5 | 12 | 11 | 1 | 4 | 2 | 5 | 1 | 0 | 12 | 0 |
| Total | 1 | 8 | 13 | 15 | 2 | 4 | 8 | 12 | 5 | 1 | 33 | 1 |
| All cases | 9 | | 28 | | 6 | | 20 | | 6 | | 34 | |

SILs, squamous intraepithelial lesions; MIC microinvasive carcinoma; HPV, human papillomavirus; +, positive; −, negative; high risk, HPV type 16, 18 and 33; low risk, HPV type 11; unknown, positive for generic HPV DNA probe but not HPV type 6, 11, 16, 18, 31 and 33 specific probes. Benign cervical lesions include cervicitis, squamous metaplasia, polyp, atypical gland and exophytic condyloma.

 © Cancer Research Campaign 1998

### MATERIALS AND METHODS

### Cervical specimens

Samples were obtained by punch biopsy of lesions under direct visualization or under colposcopic examination. Specimens were divided into two parts. The first part was submitted to routine histological examination. For the second part, a sample was further dissected and divided into two equal quantities. One part was placed in collagenase solution (collagenase 200 u $ml^{-1}$; RPMI buffer) for 24 hs. After enzymatic separation, epithelial cells and adjacent connective tissue were harvested separately, snap frozen and stored at –80°C until further use (Freshney 1987). The other part was immediately stored at –80°C for subsequent TRAP analysis. The small sample was kept at –80°C for subsequent TRAP analysis only.

Histological examination of all samples was performed by one pathologist (SN). The histological diagnoses distinguished between normal epithelium, benign lesions (such as cervicitis, polyp, exophytic condyloma, atypical glands and squamous metaplasia), low-grade SILs, high-grade SILs, microinvasive and invasive carcinoma. In cases of invasive carcinoma, only those classified as squamous-cell lesions were used for further analysis.

### TRAP assay

TRAP was performed as described previously (Kim et al. 1994; Wright et al. 1995). Briefly, each frozen tissue was first washed in 500 µl of ice-cold phosphate-buffered saline (PBS), then homogenized in 20–200 µl of ice-cold CHAPS lysis buffer according to the size of sample with a manual homogenizer. The collagenase-treated specimens were washed with ice-cold PBS and treated with lysis buffer without homogenization. After 30 min incubation on ice, the lysate was centrifuged at 14 000 ***g*** for 30 min at 4°C. The supernatant was aliquoted, flash frozen in liquid nitrogen and stored at –80°C until further analysis. The pellets were kept for subsequent HPV analysis.

Epstein–Barr virus-transformed human lymphocytes (American Type Culture Collection Cell Line, B958) were used as positive controls. The PCR-based assay was carried out in a 50-µl reaction mixture containing 2µg of the extracted protein, 20 mM Tris-HCl (pH 8.3), 1.5 mM magnesium chloride, 63 mM potassium chloride, 0.005% Tween-20, 1 mM EGTA, 50 µM dGTP, dATP and dTTP, 5 µM dCTP, 0.1 µg of TS, 1 µg of T4g32 protein, 4 µl of [α-$^{32}$P]dCTP (10 µCi $µl^{-1}$, 3000 Ci $mmol^{-1}$), 2 units of *Taq* polymerase, $5 \times 10^{-18}$ g of internal telomerase assay standard (ITAS) in a 0.5-ml tube, containing 0.1 µg of 5′-[CCCTTA]$_3$CCCTAA-3′ (CX) sealed at the bottom by a wax barrier.

After 10 min incubation at 23°C to allow telomerase-mediated extension of the 5′-AATCCGTCGAGCAGAGTT-3′ (TS) primer, the reaction mixture was then subjected to 30 PCR cycles at 94°C for 1 min, 50°C for 1 min and 72°C for 1 min. Aliquots (5 µl) of the PCR products were analysed on an 8% non-denaturing polyacrylamide gel. The gel was subsequently exposed to a Kodak XAR-5 X-ray film at –70°C with an intensifying screen. Duplicated assays were performed on all samples with RNAase pretreatment at a final concentration of 0.05 mg $ml^{-1}$ for 10 min at room temperature. The positive results were compared with the telomerase activity of ten, 100 and 1000 cells of B958.

The samples exhibiting negative ITAS were subjected to two-step TRAP (JW Shay personal communication). The first part consisted of TS primer extension with a 50-µl reaction mixture containing 2µg of the extracted protein, 20 mM Tris-HCl, 1.5 mM $MgCl_2$, 63 mM KCl, 0.005% Tween-20, 1 mM EGTA, 50 µM of each dNTP, 0.1 µg of TS, 1 µg of T4g32 protein and DEPC $H_2O$. The reaction tube was incubated in the thermocycler at 23°C for 15 min. The product was then subjected to standard phenol–chloroform DNA extraction and ethanol precipitation. The precipitate was dissolved and amplified in the second part. The second part reaction mixture was the same as in the non-modified TRAP with the incubation at 23°C for 15 min omitted. The amplification cycle was the same as the original protocol.

Positive assays were reconfirmed with and without RNAase pretreatment with newly described primers (Kim et al. 1997). The PCR-based assay was carried out in a 50-µl reaction mixture containing 2µg of the extracted protein, 20 mM Tris-HCl (pH 8.3), 1.5 mM $MgCl_2$, 63 mM KCl, 0.005% Tween-20, 1 mM EGTA, 50 µM dGTP, dATP and dTTP, 5 µM dCTP, 0.1 µg of TS, 1 µg of T4g32 protein, 4 µl of [α-$^{32}$P] dCTP (10 µCi $µl^{-1}$, 3000 Ci $mmol^{-1}$), 2 units of *Taq* polymerase and DEPC $H_2O$ in a 0.5-ml tube, containing 0.1 µg of 5′-GCGCGG[CTTACC]$_3$CTAACC-3′ (ACX) sealed at the bottom by a wax barrier.

After 10 min incubation at 23°C to allow telomerase-mediated extension of the TS primer, the reaction mixture was subjected to 30 PCR cycles at 94°C for 1 min, 50°C for 1 min and 72°C for 1 min. Aliquots (5 µl) of the PCR products were analysed on an 8% non-denaturing polyacrylamide gel. The gel was subsequently exposed to a phosphor screen and the bands were visualized on a PhosphorImager using Image Quant software (Molecular Dynamics, Sunnyvale, CA, USA).

### HPV PCR

Pellets derived from the previous extraction with CHAPS lysis buffer were subjected to standard DNA extraction (Maniatis et al. 1989). The DNA was used for subsequent HPV PCR. Control HPV DNA used in the amplification was obtained from Hela cell lines.

L1 and E6 amplification were performed as previously described (Resnick et al. 1990; Bauer et al. 1991) with 1 µl of each specimen in a 50-µl reaction mixture. Each L1 amplification reaction contained 25 pmol each of the L1 degenerate primers MY11 and MY09 and 2.5 pmol each of β-globin primers GH20 and PC04. The E6 reactions contained 5 pmol of WD72, WD66 and WD154, 20 pmol of WD67 and WD76. Both reactions were performed in a buffer containing 50 mM KCl, 10 mM Tris (pH 8.3), 4 mM $MgCl_2$, 200 µM of each dNTP and 1.25 units of *Taq* polymerase and subjected to 40 amplification cycles. Each cycle was performed at 95°C for 1 min, at 55°C for 1 min and at 72°C for 2 min. An additional 5-min final elongation cycle at 72°C was included. The PCR reactions were then separated by 2% agarose gel electrophoresis and visualized under UV illumination after ethidium bromide staining.

### Dot hybridization of PCR products

L1 and E6 type-specific probes and consensus L1 probes were used for HPV typing. Positive controls of HPV type 6, 11, 16, 18, 31 and 33 from each PCR amplification were included. Products obtained from each PCR reaction were heated to 95°C and thereafter 1 volume of 20 × SSC was added. Aliquots of 2 µl were applied to a Hybond-N$^+$ nylon membrane (Amersham, Life Science) prewetted in denaturing solution (1.5 M NaCl, 0.5 M

© Cancer Research Campaign 1998

**Figure 2** Telomerase activity in benign cervical lesions using the new set of primers (+ and –, with and without RNAase treatment respectively). Extracts of Epstein–Barr virus transformed human lymphocyte cell lines were used as positive control. Serial dilution of $10^3$, $10^2$ and ten cells represented levels of activity of the enzyme. Samples C58, C109, C157 and C187 were cervicitis, atypical gland, squamous metaplasia and polyp respectively

**Table 2** Telomerase expression level, HPV status and cervical lesions

| | Benign cervical lesions | | SILs | | INV CA | |
|---|---|---|---|---|---|---|
| **Telomerase activity** | **HPV+** | **HPV–** | **HPV+** | **HPV–** | **HPV+** | **HPV–** |
| Positive | 1 | 12 | 7 | 3 | 25 | 13 |
| <100 | 1 | 10 | 2 | | 4 | 1 |
| 100–1,000 | | 1 | 4 | 3 | 5 | 3 |
| >1000 | | 1 | 1 | | 16 | 9 |
| Negative | 4 | 11 | 7 | 9 | 2 | 0 |
| Total | 28 | | 26 | | 40 | |

HPV+, positive for human papillomavirus DNA; HPV–, negative for human papillomavirus DNA; INV CA, invasive carcinoma.

© Cancer Research Campaign 1998

NaOH). The membranes were transferred to a filter paper soaked in neutralizing solution (1.5 M NaCl, 0.5 M Tris-HCl pH7.2, 0.001 M EDTA) for 1 min. The membranes were air dried at room temperature, soaked in 0.4 M NaOH for 20 min for fixation and washed with 5 × SSC. Prehybridization at 65°C for 1 h was carried out using 6 × SSC, 5 × Denhardt's solution, 0.5% SDS, 100 µg of single-stranded sheared salmon sperm DNA per millilitre. Replicate membranes were separately hybridized with denatured $^{32}$P-labelled, type-specific probes in prehybridizing solution for 1 h at 55°C. Probes WD170 required hybridization at 45°C. Filters were rinsed briefly in 2 × SSC and 0.1% SDS at room temperature and then twice for 10 min at 45°C (WD170), 50–52°C (WD132, RR1 and RR2), 55–56°C (WD103, WD165 and WD166), 56–57°C (consensus L1, MY12/13, WD126, WD128, MY16 and WD133/134) or 58–59°C (MY14 and WD74). The membranes were subjected to autoradiography using a Kodak XAR-5 film. Results of L1 and E6 dot blots were scored independently. Duplicate filters were prepared for all specimens.

### Data and statistical analysis

Data in each part, histology, TRAP assay, HPV PCR and typing, were collected in a double-blind fashion until further analysis. The chi-square test was used to compare the results obtained from telomerase analysis with those of HPV and pathological parameters.

### RESULTS

### Telomerase activity in cervical lesions

In the present study, we analysed nine samples of normal controls, 28 of benign cervical lesions, six of low-grade SILs, 20 of high-grade SILs, six of microinvasive carcinoma (MIC) and 34 of squamous-cell carcinoma of the cervix. The benign cervical lesions included 19 cases of cervical cervicitis, four cases of squamous metaplasia, two cases of atypical gland, two cases of polyps and one case of exophytic condyloma. Telomerase was found activated in five cases of MIC and 33 cases of squamous-cell carcinoma. A decreased incidence of expression was identified in SILs, two out of six cases of low-grade SILs and 8 out of 20 cases of high-grade SILs (Figure 1 and Table 1). Interestingly, 13 out of 28 cases of benign cervical lesions, eight cases of cervicitis, two cases of atypical glands, two cases of squamous metaplasia and one case of polyps, and one out of nine cases of normal controls were positive for telomerase activity. (Figure 2 and Table 1)

Because the cervical lesions showed variations regarding the intensity of the TRAP signals, we compared the intensity of the ladder signals with serial dilutions of the cell line B958, 100 and 1000 cells respectively. A gradual increment of TRAP intensity was observed that varied in direct proportion to the number of the immortalized cells tested. We then semiquantitatively compared the density of each positive TRAP result with the serially diluted B958 cell line (Table 2). The dosage of telomerase activity was then recorded as <100, 100–1000, or >1000, indicating whether the intensity of those bands was less than 100, or between 100 and 1000 or more than 1000 B958 cells respectively. The variation in expression of telomerase has been detected in all types of cervical lesions. Nevertheless, in general the invasive cancers expressed the highest telomerase activity and the benign lesions and normal cervical tissues expressed the lowest. This comparison was valid in both HPV-positive and -negative groups.

In order to identify whether telomerase expression originated from epithelium or activated lymphocytes in connective tissue, collagenase treatment was applied to some SILs and benign lesions. In the case of most lesions, a higher level of telomerase activity was detected in epithelial tissues. In addition, there were four cases of SILs and one case of cervicitis where we could ascribe the telomerase activity exclusively to the epithelium. Hence, detectable telomerase activity should be located within the epithelial layer of the cervix (Figure 3).

### Telomerase activity, HPV status and cervical lesions

This study identified HPV in 24%, 54% and 68% of benign cervical lesions, SILs, and invasive carcinoma respectively. Among these HPV-positive samples, intermediate/high-risk HPV, type 16, 18 or 33, was detected in 36 out of 48 cases. Low-risk HPV was identified only in one case of low-grade SILs. The remaining samples were not positive for type 6, 11, 16, 18, 31 and 33 specific probes (Table 1 and Figure 4).

Although HPV was detectable in the cervices of all group tested, a different frequency of positive TRAP assay was observed when comparing the groups (Table 2). Whereas 93% of HPV-positive cancers showed telomerase expression, only 50% of SILs expressed the enzyme ($P<0.005$; cancer vs SILs). Interestingly, a significant distinction regarding telomerase activity was also observed between SILs and cancer cases in the HPV-negative group ($P<0.001$). This suggests that telomerase may be activated during late multistep carcinogenesis in both groups.

As for benign cervical lesions, no significant correlation of telomerase activity with the HPV status ($p>0.5$) became apparent. Whereas 20% of HPV-positive cases exhibited telomerase activity, 52% of the HPV-negative group were telomerase positive in the TRAP assay. This may imply that HPV infection does not lead to telomerase activation in benign cervical lesions (Table 2).

### DISCUSSION

Telomerase is a ribonucleoprotein enzyme that synthesizes telomeres, the specialized structures containing unique simple repetitive sequences (TTAGGG in vertebrates) at the end of chromosomes (Blackburn 1991; Feng et al, 1995). The enzyme activity provides the means to compensate for the end replication problem and thus expression of telomerase might be a crucial factor allowing cells to proliferate indefinitely (Counter et al, 1992). Once thought to be cancer-cell specific, it is becoming apparent that many proliferating normal cells express telomerase activity at a lower level. Among these are human activated lymphocytes and several types of epithelium (Taylor et al, 1996; Yasumoto et al, 1996; Engelhardt et al, 1997). This study performed on the cervix demonstrated similar findings. A low level of telomerase activity was detectable in normal cervical tissues and benign lesions and may be necessary for their physiological proliferation. On the other hand, the pattern of telomerase expression in SILs and carcinoma cases indicates that telomerase is involved during cancer development and activated during the late stage of multistep carcinogenesis.

In order to identify whether and how HPV is associated with telomerase activity, several cervical samples were tested by means of TRAP assay and HPV DNA detection using L1 and E6 primers. Both techniques are very specific and sensitive methods. The TRAP assay could detect telomerase activity from as few as ten immortal cells. The new primer set, the internal control and two-step reactions

© Cancer Research Campaign 1998

**Figure 3** Telomerase activity in epithelium of cervical tissues (+ and –, with and without RNAase pretreatment respectively). ITAS, internal telomerase assay standard. Extracts of Epstein–Barr virus transformed human lymphocyte cell lines were used as positive control. Serial dilution of $10^3$, $10^2$ and ten cells represented levels of activity of the enzyme. CHAPS lysis buffer (B) was used as negative control. T represents tissue before collagenase treatment. E and C represent epithelium and connective tissues after collagenase treatment respectively. C58 was cervicitis and C54 was high-grade SILs helped solve problems regarding false-positive and negative-results due to primer dimers and PCR inhibitors respectively. In cases of HPV detection, consensus and type-specific oligonucleotide probes used in hybridization analysis of L1 amplification products were shown to detect as few as ten copies of HPV of many known and more than 25 novel HPV types (Resnick et al 1990). The second consensus primer set was designed to amplify a 240-bp region of the *E6* gene and helped improve the analysis of low-quality DNA (Resnick et al 1990). Thus, using these two methods should be appropriate to compare the telomerase activity between HPV-positive and -negative groups.

Our data showed that the presence of high-risk HPV was not associated with telomerase activation in benign cervical lesions. Only 20% of HPV-positive benign cervical lesions, four cases of high-risk and one case of unknown type, expressed telomerase. On the contrary, 52% of the HPV-negative group showed enzyme activity. Although this data did not imply that HPV could not activate telomerase in benign cervical lesions, HPV does not play a crucial role and other mechanism(s) might be involved.

**Figure 4** Dot-blot hybridization for HPV 16 probe. Lanes a1–f1 were positive controls using purified plasmids of HPV type 6, 11, 16, 18, 31 and 33 respectively. Lane g1 was extracted DNA from HeLa cells. Lanes a2–g2 were invasive carcinoma lesions. Lanes a3–d3 and e3–g3 were cervicitis and normal cervix respectively

© Cancer Research Campaign 1998

One of the most crucial parts played by HPV in cervical carcinogenesis is activation of telomerase. It will be interesting to explore whether HPV infection alone or additional genetic alterations are required for telomerase activation during cervical carcinogenesis. Several studies performed on the transforming activity of HPV have shown the capability of high-risk HPV E6 to transform cells to the immortalized state. One study on human keratinocyte retrovirus-mediated transduction found that the E6 protein alone was sufficient for early telomerase activation (Klingelhutz et al 1996; Stoppler et al 1997). However, another study on human keratinocytes transfected with full-length, high-risk HPV has shown additional genetic events to be required for increased levels of telomerase expression (Steenbergen et al, 1996). Our data showed that 50%, 80% and 95% of HPV-positive SILs, MIC and squamous-cell cancer expressed telomerase respectively. This provided evidence that the transforming activity of high-risk-type HPV did not encompass telomerase activation at the initiation of cervical carcinogenesis, but might be triggered by subsequent genetic aberrations at some later stage.

Previous studies demonstrated that telomerase activity was detectable in cervical scrapings, suggesting that the TRAP assay is a useful screening method for cervical lesions, especially when combined with a Pap smear test (Kyo et al 1997). However, our results demonstrated that more than 40% of benign lesions and normal cervical tissues were positive in the TRAP assay, regardless of their HPV status. This may limit the usefulness of this application. Nevertheless, as a very high frequency of telomerase expression was observed in the early invasive lesions, this enzyme activity may constitute a biomarker regarding malignant transformation or invasiveness. It may be worth pursuing the clinical usefulness of this molecular marker in the future.

### ACKNOWLEDGEMENTS

We are indebted to all staff in the Department of Obstetrics and Gynecology and the Radiology Unit, Department of Radiology, Chulalongkorn University Hospital, who provided all clinical samples; to Ms Saengsoon Trirekapan, Mr Wichai Porntanakasem and Ms Sairoong Sakdikul for technical assistance; to Drs Parvapan Bhattarakosol and Virapong Lulitanond for providing purified HPV plasmid; to Dr Jerry W Shay for technical advice; and to Dr Karol Sikora, Ms Petra Hirsch and Dr Henry Wilde for their critical review of the manuscript. This work was supported by the Molecular Biology Project, Faculty of Medicine, Chulalongkorn University, and the Thailand Research Funds.

### REFERENCES

Anderson S, Shera K, Ihle J, Billman L, Goff B, Greer B, Tamini H, McDougall J and Klingelhutz A (1997) Telomerase activation in cervical cancer. *Am J Pathol* **15**: 25–31

Bauer HM, Ting Y, Greer CE, Chambers JC, Tashiro CJ, Chimera J, Reingold A and Manos MM (1991) Genital human papillomavirus infection in female university students as determined by a PCR-based method. *JAMA* **265**: 472–477

Blackburn EH (1991) Structure and function of telomeres. *Nature* **350**: 569–573

Burger MPM, Hollema H, Pieters WJLM, Schroder FP and Quint WGW (1996) Epidermiological evidence of cervical intraepithelial neoplasia without the presence of human papillomavirus. *Br J Cancer* **73**: 831–836

Bosch FX, Manos MM, Munoz N, Sherman M, Jansen AM, Peto J, Schiffman MH, Moreno V, Kurman R, Shah KV and International Biological Study on Cervical Cancer Study Group (1995) Prevalence of human papillomavirus in cervical cancer: a worldwide perspective. *J Natl Cancer Inst* **87**: 796–802

Counter CM, Avilion AA, LeFeuvre CE, Stewart NG, Greider CW, Harley CB and Bacchetti S (1992) Telomere shortening associated with chromosome instability is arrested in immortal cells which express telomerase activity. *EMBO J* **11**: 1921–1929

DiPaolo JA, Popescu NC, Alvarez L and Woodworth CD (1993) Cellular and molecular alterations in human epithelial cells transformed by recombinant human papillomavirus DNA. *Crit Rev Onc* **4**: 337–360

Engelhardt M, Kumar R, Albanell J, Pettengell R, Han W and Moore MA (1997) Telomerase regulation, cell cycle, and telomerase stability in primitive hematopoietic cells. *Blood* **90**: 182–193

Feng J, Funk WD, Wang S, Weinrich SL, Avilion AA, Chiu C, Adams RR, Chang E, Allsopp RC, Yu J, Le S, West MD, Harley CB, Andrews WH, Greider CW and Villeponteau B (1995) The RNA component of human telomerase. *Science* **269**: 1236–1241

Freshney RI (1987) *Culture of Animal Cells a Manual of Basic Technique*. John Wiley & Son New York **2**: 122–124

Kim NW and Wu F (1997) Advances in quantification and characterization of telomerase activity by the telomeric repeat amplification protocol (TRAP). *Nucleic Acids Res* **25**: 2595–2597

Kim NW, Piatyszek MA, Prowse KR, Harley CB, West MD, Ho PL, Coviello GM, Wright WE, Weinrich SL and Shay JW (1994) Specific association of human telomerase activity with immortal cells and cancer. *Science* **266**: 2011–2015

Klingelhutz AJ, Foster SA and McDougall JK (1996) Telomerase activation by the E6 gene product of human papillomavirus type 16. *Nature* **380**: 79–82

Kyo S, Takakura M, Ishikawa H, Sasagawa T, Satake S, Tateno M and Inoue M (1997) Application of telomerase assay for the screening of cervical lesions. *Cancer Res* **57**: 1863–1867

Lundberg GD (1989) The 1988 Bethesda system for reporting cervical/vaginal cytological diagnoses. *JAMA* **262**: 931–934

Maniatis T, Fritsch EF and Sambrook J (1989) *Molecular Cloning: A Laboratory Manual*, 2nd edn. Cold Spring Harbor Laboratory: Cold Spring Harbor, NY

Mutirangura A, Supiyaphun P, Trirekapan S, Sriuranpong V, Sakuntabhai A, Yenrudi S and Voravud N (1996) Telomerase activity in oral leukoplakia and head and neck squamous cell carcinoma *Cancer Res* **56**: 3530–3533

Pao CC, Tseng CJ, Lin CY, Yang FP, Hor JJ, Yao DS and Hsueh S (1997) Differential expression of telomerase activity in human cervical cancer and cervical intraepithelial neoplasia lesions. *J Clin Oncol* **15**: 1932–1937

Resnick RM, Cornelissen MTE, Wright DK, Eichinger GH, Fox HS and Manos MM (1990) Detection and typing of human papillomavirus in archival cervical cancer specimens by DNA amplification with consensus primers. *J Natl Cancer Inst* **82**: 1477–1484

Schiffman MH, Bauer HM, Hoover RN, Glass AG, Cadell DM, Rush BB, Scott DR, Sherman ME, Kurman RJ, Wacholder S, Stanton CK and Manos MM (1993) Epidemiologic evidence showing that human papillomavirus infection causes most cervical intraepithelial neoplasia. *J Natl Cancer Inst* **85**: 958–964

Shay JW and Gazdar AF (1997) Telomerase in the early detection of cancer (review). *J Clin Pathol* **50**: 106–109

Stoppler H, Hartmann DP, Sherman L and Schlegel R (1997) The human papillomavirus type 16 E6 and E7 oncoproteins dissociate cellular telomerase activity from the maintenance of telomere length. *J Biol Chem* **272**: 13332–13337

Steenbergen RDM, Walboomers JMN, Meijer CJLM, van der Raaij-Helmer EMH, Parker JN, Chow LT, Broker TR and Snijders PJF (1996) Transition of human papillomavirus type 16 and 18 transfected human foreskin keratinocytes towards immortality: activation of telomerase and allele losses at 3p, 10p, 11q and/or 18q. *Oncogene* **13**: 1249–1257

Taylor RS, Ramirez RD, Ogoshi M, Chaffins M, Piatyszek MA and Shay JW (1996) Detection of telomerase activity in malignant and nonmalignant skin conditions *J Invest Dermatol* **106**: 759–765

Whelan SL, Parkin DM and Masuyer E (1990) *Pattern of Cancer in Five Continents* Lyon: International Agency for Research on Cancer

Wright WE, Shay JW and Piatyszek MA (1995) Modifications of a telomeric repeat amplification protocol (TRAP) result in increased reliability, linearity and sensitivity. *Nucleic Acids Res* **23**: 3794–3795

Yasumoto S, Kunimura C, Kikuchi K, Tahara H, Ohji H, Yamamoto H, Ide T and Utakoji T (1996) Telomerase activity in normal human epithelial cells. *Oncogene* **13**: 433–439

Zheng PS, Iwasaka T, Yokoyama M, Nakao Y, Pater A and Sugimori H (1997) Telomerase activation in in vitro and in vivo cervical carcinogenesis. *Gynecol Oncol* **66**: 222–226

© Cancer Research Campaign 1998